# *PLOS Neglected Tropical Diseases* 2017 Reviewer and Editorial Board Thank You

**DOI:** 10.1371/journal.pntd.0006359

**Published:** 2018-03-15

PLOS and the *PLOS Neglected Tropical Diseases (NTDs)* team would like to express our appreciation for our associate editors, guest editors, and reviewers who contributed to the peer-review process this past year. We are indebted to our volunteers who generously give their time and expertise to thoroughly review research and advance Open Access. In 2017, *PLOS NTDs* received the help of 266 Editorial Board members and 165 guest editors to curate 1,567 submissions. Along with the participation of 2,664 reviewers, we were able to publish 774 articles with meaningful and impactful results.

The names of our 2017 editors that handled submitted manuscripts appear in the Supporting Information as S1 Editor List and as S1 Guest Editor List. Our reviewers appear in the Supporting Information as S1 Reviewer List. With genuine gratitude, we thank you all for your dedicated support of *PLOS Neglected Tropical Diseases* and our efforts to promote Open Science, thereby contributing to the scientific community as a whole. Thank you all.

## Supporting information

S1 Editor List Alphabetical list of 2017 *PLOS NTDs* Editorial Board Members. (PDF)

S1 Guest Editor List Alphabetical list of 2017 *PLOS NTDs* Guest Editors. (PDF)

S1 Reviewer List Alphabetical list of 2017 *PLOS NTDs* peer reviewers. (PDF)
