# Correction: *PLOS Neglected Tropical Diseases* 2017 Reviewer and Editorial Board Thank You

**DOI:** 10.1371/journal.pntd.0006424

**Published:** 2018-04-13

**Authors:** The PLOS Neglected Tropical Diseases Staff

## Notice of republication

This article was republished on March 29, 2018, to correct errors in the statistics as well as the editor and guest editor lists. The publisher apologizes for the errors. Please download this article again to view the correct version. The originally published, uncorrected editor lists and the republished, corrected editor lists are provided here for reference.

## Supporting information

S1 Editor List Originally published, uncorrected editor list. (PDF)

S1 Guest Editor List Originally published, uncorrected guest editor list. (PDF)

S2 Editor List Republished, corrected editor list. (PDF)

S2 Guest Editor List Republished, corrected guest editor list. (PDF)

## References

[1] (2018) *PLOS Neglected Tropical Diseases* 2017 Reviewer and Editorial Board Thank You. PLoS Negl Trop Dis 12(3): e0006359 https://doi.org/10.1371/journal.pntd.0006359 29543800

