# Supplementary material for: PLOS Neglected Tropical Diseases 2017 Reviewer and Editorial Board Thank You
Source: PLoS Negl Trop Dis. 2018 Mar 15;12(3):e0006359. doi: 10.1371/journal.pntd.0006359 (PMC5854236; doi:10.1371/journal.pntd.0006359)
Supplement: S1 Guest Editor List — (PDF) [file pntd.0006359.s002.pdf]

PLOS Neglected Tropical Diseases would like to thank all those who served as Guest Associate Editors in 2017:

Adly M. M. Abd-Alla
David G. Addiss
Sitara S. R. Ajjampur
Adam Akullian
Igor C. Almeida
Cheryl Andam
Andrea Angheben
Charles Apperson
Philip M. Armstrong
Michael Benjamin Arndt
Geoffrey M. Attardo
Stephen W. Attwood
Guillermina Avila
Subash Babu
Kelly K. Baker
Gad Baneth
Mazin Barry
Lyric C. Bartholomay
Daniella C. Bartholomeu
Steve Bellan
Joshua B. Benoit
Stuart D. Blacksell
Mariangela Bonizzoni
Bradley R. Borlee
Christian Bottomley
Lilian Lacerda Bueno
Dora Buonfrate
Amaya Lopez Bustinduy
Juan J. Calvete
Suzy J. Campbell
Jong-Yil Chai
Mitali Chatterjee
Rebecca C. Christofferson
Julie Clennon
Laurent Coudeville
Robert Cowie
Thomas C. Darton
Albert Descoteaux
Gregory Deye
George Dimopoulos
Sheila M. Donnelly
Jennifer A. Downs
Susanna Jane Dunachie
Gregory D. Ebel
Anna-Bella Failloux
Nicholas Feasey
Peter U. Fischer
Agnes Fleury
Dina M. Fonseca
Brett M. Forshey
Carlos Franco-Paredes
Michael French
Isaac Chun-Hai Fung
Sreenivas Gannavaram
Michael W. Gaunt
Andréa Gazzinelli
Anna Geretti
Robert H. Gilman
Eva Gluenz
Federico Gobbi
Ángel González
Alemayehu A. Gorfe
Luigi Gradoni
Ikram Guizani
Margaret Gyapong
Abdulrazaq G. Habib
Jo E. B. Halliday
Kate A. Halton
Billy Harnett
Mary Hayden
Wayne Hodgson
Michael R. Holbrook
Peter Horby
Marc P. Hübner
Amit Huppert

Andrew Paul Jackson
Emmitt R. Jolly
William B. Klimstra
Amy D. Klion
Alain Kohl
Peter J. Krause
Ulrich Kuch
Ruth Kutalek
Juan Pedro Laclette
Poppy H. L. Lamberton
Andres G. Lescano
Michael D. Lewis
Lu-Yun Lian
Benjamin Liebeskind
Ulisses Gazos Lopes
Philip T. LoVerde
Sheila Lukehart
Calum N. L. Macpherson
Louis Maes
Mathieu Maheu-Giroux
Pablo Maravilla
Florian Marks
Hazel Elizabeth Mccullough
Henry John McSorley
Arianna R. Means
Rojelio Mejia
Fela Mendlovic
Edwin Michael
Paul Mireji
Edward Mitre
Susan Moore
Alessandra Morassutti
Eric Mossel
Hira L. Nakhasi
Steven J. Norris
Brandon Ogbunugafor
Juan Olano
Fabiano Oliveira
Martin Olivier
Antonio Osuna
Anna Papa
Shama Parveen
Patricia Pavlinac
Valerie A. Paz-Soldan
Pamela Marie Pennington
Townsend Peterson
William A. Petri Jr.
Hendrik Poinar
Edoardo Pozio
Maia A. Rabaa
Vedantam Rajshekhar
Ramesh Ratnappan
Simon Rayner
Nicholas G. Reich
Robert C. Reiner
Anne Rimoin
Isabel Rodriguez-Barraquer
Stephen John Rogerson
Mara Cecilia Rosenzvit
Syamal Roy
Masayuki Saijo
Elda Sanchez
Euzenir Nunes Sarno
Manuel Schibler
Donald S. Shepard
Sujan Shresta
Mar Siles-Lucas
Rachel E. Simmonds
Cameron P. Simmons
Fabrice Simon
Darci Smith
Wilma A. Stolk
Michel Tibayrenc
Kirkby D. Tickell
Paul Robert Torgerson
Alfredo G. Torres
Rebecca Justine Traub
Lance Turtle
Johan van Griensven
Lisette van Lieshout
Nikos Vasilakis
Wei-Kung Wang
Samuel Wanji
Guilherme L. Werneck
Mark L. Wilson
Andrea Winkler

Laila Woc Colburn
Adrian J. Wolstenholme
Chaoyang Xue
Yoshihisa Yamano
