# Supplementary material for: PLOS Neglected Tropical Diseases 2017 Reviewer and Editorial Board Thank You
Source: PLoS Negl Trop Dis. 2018 Mar 15;12(3):e0006359. doi: 10.1371/journal.pntd.0006359 (PMC5854236; doi:10.1371/journal.pntd.0006359)
Supplement: S1 Reviewer List — (PDF) [file pntd.0006359.s003.pdf]

*PLOS Neglected Tropical Diseases* would like to thank all those who reviewed on behalf of the journal in 2017:

John Aaskov
Fernando Abad-Franch
Syed Abbas
Mohamed Faizal Abdul-Careem
Eniola Abe
Mesfin Abebe
Claudia Abeijon
David Abraham
Asha Abraham
Abdinasir Abubakar
Nicole Achee
Mark Achtman
Mercy Ackumey
Luis Actis
Ishag Adam
Linda Adams
Emily Adams
David Addiss
Wasiu Olalekan Adebimpe
Ahmed Adeel
Oyelola Adegboye
Adebiyi Adesina
Adedoyin Adesina
Daniel Adesse
Babatunde Adewale
Kazeem Adeyemi
Bishwa Adhikari
Haritha Adhikarla
Abass Adigun
Philip Adongo
Moses Adriko
Vincent Adung'a
Toni Aebischer
Eve Afonso
Luis Afonso
Philippe Afonso
Farhat Afrin
Suneth Agampodi
Natasha Agramonte
Maira Aguiar
Anwar Ahmed
Osama Ahmed Hassan Ahmed
Collins Ahorlu
Steven Aird
Okan Akhan
Rama Akondy
George Akpede
Hannah Akuffo
Kalichamy Alagarasu
Pietro Alano
Al-Sayed R. Al-Attar
Marco Albonico
Gabriel Alcoba
Gabriel Alcoba
Pedro Alcolea
Jared Aldstadt
Hassan Al-Emran
Neal Alexander
Jackeline Alger
Asad Ali
Ibne Ali
Nahid Ali
Mohammad Ali
Matthew Aliota
Amer Al-Jawabreh
S.M. Alkmim-Oliveira
Brian Allan
Fiona Allan
Lee-Ann Allen
Stephen Allen
Denise Allen
Pascale Allotey
David Allred
Shihana Almatruk

Thiago Almeida
Igor Almeida
Alzira Almeida
Roque Almeida
Hesham Al-Mekhlafi
Guillermo Alonso
Cristina Alonso-Vega
Haoues Alout
Sam Alsford
Bulent Alten
Thomas Althaus
Barry Alto
Kishore Alugupalli
Vanina Alvarez
Cristian Alvarez Rojas
Maria Júlia Alves
Ahmed Aly
Hussein Aly Ibrahim
Gaya Amarasinghe
Valdir Amato
Kei Amemiya
Brian Amman
Brian Amman
Yaw Amoako
Francis Amrit
Neil Anderson
Lisa Anderson
Burt Anderson
Roy Anderson
Jennifer Anderson
Deborah Anderson
Tavis Anderson
John Anderson
Hélida Andrade
Luciana Andrade
Jose Dilermando Andrade-Filho
Jason Andrews
Rodrigo Angerami
Andrea Angheben
Fernanda Anibal
Andrey Anisimov
Paulo Renato Antas
Marina Antillón
Spinello Antinori
Styliani Antonara
Karim Aoun
Juan Aparicio
Ruslan Aphasizhev
Charles Apperson
Grace Appiah
Solomon Aragie
Byron Arana
Jackson Araújo
Jacqueline Araujo Fiuza
Bruno Arca
Ricardo Arcencio
Ana Paula Arez
Ariaranee Ariaratnam
Kevin Ariën
Shumaila Arif
Yuji Arimatsu
María Arnaiz
Raffi Aroian
Carlos Arregui
Muhammad Imran Arshad
Jose Arteaga Bejarano
Marios Arvanitis
Sassan Asgari
Ben Ashby
Anthony Ashton
Kingsley Asiedu
Emmanuel Assampong
Ricardo Ataide
Badolo Athanase
Daniel Athanazio
Robert Atmar
Geoffrey Attardo
Nicolas Aubrey
Sarah Auburn
Albert Auguste
Guillermina Avila
Tatjana Avsic-Zupanc
Abdu Azad
Taichi Azuma
Simon Babayan
Alexandru Babes

Subash Babu
Bontha Babu
Arthur Baca
Horacio Bach
Michael Bachman
Jacqueline Badaki
Alan Baer
Oliver Baerenbold
Eduardo Bagagli
Fahimeh Bagheri Amiri
Saiku Bah
J. Baird
Sylvain Baize
Anthony Baker
Tamas Bakonyi
Shirish Balachandra
Valdir Balbino
Thomas Balenghien
Jacob Ball
Alberto Baly
Gad Baneth
Mary Lynn Baniecki
Ashley Banyard
Changjun Bao
R. A. Barata
Bridget Barber
David Barbosa
Alan Barbour
Thales Barçante
Stefano Barco
Beatrice Barda
Avner Bar-Hen
Andrew Barnes
Yves Barogui
Veronica Barragan
Aldina Barral
Amilton Barreira
Luis Barreiro
Janine Barrett
Michael Barrett
Alan Barrett
Jean-Mathieu Bart
Luther Bartelt
Richard Bartfai
Lyric Bartholomay
Daniella Bartholomeu
Sarah Bartsch
Chris Bass
Leonardo Bastos
Carolina Batista
Sina Bavari
Himmatrao Bawaskar
Chiara Bazzocchi
Mathew Beale
Andrea Beaton
Baltazar Becerril
Namseon Beck
Sören Becker
Stefanie Becker
Charmagne Beckett
J. David Beckham
Stéphanie Bedhomme
Jamie Bedson
Nigel Beebe
Arlyne Beeche
Brianna Beechler
Nick Beeton
Vicente Belizario
Bryan Bellaire
Hedia Bellali
Silvana Belo
Gil Benard
Mark Benbow
Jade Benjamin-Chung
Issam Bennis
Sasisekhar Bennuru
Joshua Benoit
Dennis Bente
Mary Berbee
Éric Bergeron
David Bergman
Indra Bergval
J. D. Berman
Luiz Bermudez
Kristen Bernard
Corina Beron

Nicolas Berthet
A. Bertoletti
Enrico Bertuzzo
Bernard Bett
Lucien Bettendorff
Judy Bettridge
Stephen Beverley
Sarah Bevins
Sayan Bhattacharya
Suvendra Bhattacharyya
Tapan Bhattacharyya
Mrinal Bhattacharyya
Katell Bidet Huang
Shakir Bilal
Kasahun Bilcha
Zhou Bin
Samik Bindu
Nana-Kwadwo Biritwum
Nana-Kwadwo Biritwum
Subhajit Biswas
Ashis Biswas
William Black IV
Stuart Blacksell
Carol Blair
David Blaney
Lucas Blanton
Jesse Blanton
Joerg Blessmann
Catherine Blish
Jeffrey Bloomquist
Maria Heloísa Blotta
Johannes Blum
Daniel Boakye
Boakye Boatin
Viviane Boaventura
Jes-Niels Boeckel
David Boels
Isaac Bogoch
Christine Boinett
Jan Boitz
Mariangela Bonizzoni
Adrianus Boon
Seth Bordenstein
Charissa Borja-Tabora
Anna Borlase
Irene Bosch
Christopher Bosio
Kwabena Bosompem
Monica Botelho
Jason Botten
Christian Bottomley
Pierre Bougis
David Boulware
Yap Boum II
Hervé Bourhy
Kostas Bourtzis
Samia Boussa
Michel Boussinesq
Bernard Bouteille
Jérémy Bouyer
Donald Bouyer
Natalie Bowman
Natalie Bowman
Leigh Bowman
Ross Boyce
Sebastien Boyer
Kenneth Boyer
Doug Brackney
Richard Bradbury
Mark Bradley
Molly Brady
Oliver Brady
Cynthia Braga
Sara Brant
Patricia Brasil
Aaron Brault
David Brett-Major
Gisela Bretzel
Matthew Brewer
William Brieger
James Brien
James Brien
Neima Briggs
Margo Brinton
Carlos Brisola Marcondes
Seth Britch

Carl Britto
Collette Britton
Mara Broadhurst
Norbert Brockmeyer
Claudia Brodskyn
William Brogdon
Simon Brooker
Zachary Brown
Jessica Brown
Heidi Brown
Nicholas Brown
Catherine Brown
Charles Brown
Fabrizio Bruschi
Bart Bryant
Bruno Bucheton
Anna Buczak
Sarah Buddenborg
Philip Budge
Philip Budge
Christine Budke
Ruben Bueno
Pierre Buffet
Maria Jose Buitrago
Dimitri Bulté
Donald Bundy
Andre Buret
Ben Burford
Stewart Burgess
Thorsten Burmester
Helen Burn
Mary Burtnick
Sakib Burza
Amaya Bustinduy
Peter Bütikofer
Nadia Kate Butler
Tom Butler
Ruth Butlin
Miguel Cabada
Simeon Cadmus
Sandy Cairncross
Maria Cajimat
Roberta Caldeira
A. Calderon
Ljubica Caldovic
Sabrina Calil-Elias
Theodora Calogeropoulou
Amanda Calvert
Juan Calvete
Sébastien Calvignac-Spencer
Margarita Calvo
Vitaliano Cama
Luiz Camacho
Anton Camacho
Tamara Camara
Emmanuelle Cambau
Ewan Cameron
Caroline Cameron
Federico Camicia
Cyril Caminade
Suzy Campbell
Corey Campbell
Lenea Campino
Jorge Cano
Luz Cano
Lilian Cantanhede
Paul Cantey
Shengbo Cao
Van-Mai Cao-Lormeau
José-Luis Capelo-Martínez
Malini Capoor
Margareth Capurro
Hélène Carabin
Jonathan Carapetis
Nora Cardona-Castro
Luis Cardoso
James Carey
Graeff-Teixeira Carlos
John Carlson
Elizabeth Carlton
David Carmena
Mariângela Carneiro
Elisabeth Carniel
Giovanna Carpi
Maria Cristina Carrasquilla
Julio Carrera

Lauren Carrington
Miles Carroll
Nicola Carter
K. C. Carter
Danilo Carvalho
Edgar Carvalho
Edgar Carvalho
Filipe Carvalho-Costa
Arnau Casanovas-Massana
Martin Casapia
Elizabeth Case
Kathleen Cashman
Léa Castellucci
Maria Castillo
Ricardo Castillo Neyra
Claudia Castillo-Gonzalez
Ricardo Castillo-Neyra
Osvaldo Castro Peraza
Enrique Castro-Sánchez
Yagahira Castro-Sesquen
Adriano Casulli
Andrew Catley
Eric Caumes
Luciano Cavalcanti
Laura Ceballos
Giuliano Cecchi
Maria Cecere
Igor Cestari
Catherine Cetre-Sossah
Carlos Chaccour
Joon-Seok Chae
Sebastian Chahda
Ann Chahroudi
Jong-Yil Chai
Wanpen Chaicumpa
Clemence Chako
Subhra Chakraborty
Rachel Chalmers
Jagdish Chander
David Chandler
Aileen Chang
Gwong-Jen Chang
Narisara Chantratita
Dennis Chao
Chien-Chung Chao
François Chappuis
Richelle Charles
Eric Chatelain
Raghunath Chatterjee
Mitali Chatterjee
Delphi Chatterjee
Neeraj Chauhan
Luis Fernando Chaves
Francesco Checchi
Muthu Chelvan
Bin Chen
Qijun Chen
Wei-June Chen
Rubing Chen
Jian-Ping Chen
Qingfeng Chen
Yingxi Chen
Changbin Chen
Gong Cheng
Afona Chernet
Christophe Chevillard
Claire Chewapreecha
Francisco Chiaravalloti Neto
Wei-Mei Ching
Richard Chipman
Miguel Chiurillo
Ray Cho
Nam-Hyuk Cho
Young-Jun Choi
Bruno Chomel
Sidharth Chopra
Sam Chorlton
Olivier Chosidow
Valerie Choumet
Rajib Chowdhury
Gerardo Chowell
Ashim Chowla
Henry Choy
Jean-Paul Chretien
Rebecca Christofferson
Justin Jang Hann Chu

Kaw Bing Chua
Marc Ciosi
Alexander Ciota
Cornelius Clancy
Hannah Clapham
Frederick Clasen
Christine Clayton
Julie Cleaton
Sarah Cleaveland
Jan Clements
Angela Cleveland
Steven Cobb
Camila Coelho
Luc Coffeng
Lark Coffey
Lee Cohnstaedt
Matthew Coldiron
Robert Colebunders
Ross Colgate
Philip Collender
Daniel Colley
James Collins III
Tonya Colpitts
Iñaki Comas
Anne Conan
Kathryn Conlon
Bernadette Connolly
Franz Conraths
Paul Converse
David Conway
Elizabeth Cook
Peter Cook
Piet Cools
Philip Cooper
Isabelle Coppens
Enoka Corea
Victor Corman
Helena Corrêa De Araújo
Jorge Cortés
Ileana Corvo
Gerardo Corzo
Peter Costa
Carlos Costa
Maria Costi
Jean Coulibaly
Mamadou Coulibaly
Yaya Coulibaly
Wendel Coura-Vital
Orin Courtenay
David Courtin
Iliano Coutinho-Abreu
Olivier Coux
Janet Cox-Singh
Christina Coyle
Darren Creek
Pascal Crépey
Julio Croda
Elizabeth Cromwell
Robert Cross
Ian Crozier
Israel Cruz
Angela Cruz
Karina Cucchi
Marcela Cucher
Zulma Cucunuba
Manuel Cuenca-Estrella
Liwang Cui
Richard Culleton
Edecio Cunha-Neto
Adam Cunningham
Bart Currie
Sally Cutler
Krystyna Cwiklinski
Clarissa da Costa
Andrea Da Poian
Laurent Dacheux
Alda Maria DaCruz
Akita Daigaku
Gianluca D'Amico
David Dance
Anthony Danso-Appiah
Wanderson DaRocha
Michael Darsley
Thomas Darton
Pradeep Das
Siddhartha Das

Gregory Dasch
Alexandre daSilva
Catherine Dauga
Gail Davey
Andrew Davidson
Debora d'Avila Reis
Claudia d'Avila-Levy
Stephanie Davis
April Davis
Halima Dawood
Scott Dawson
Tim Day
Carlos de Almeida
Rukie De Alwis
Solange De Castro
Reginald De Deken
Reginald De Deken
William de Glanville
Luc de Haro
Harry De Koning
Juan C. de la Torre
Marta de Lana
Giulio De Leo
Maria de Lourdes Macoris
Juliana de Menezes
Isabel de Miranda Santos
Tatiana de Moura
Geraldine De Muylder
Carlo de Oliveira
Camila de Oliveira
Adolfo de Roodt
Lalindi De Silva
Janaka de Silva
Sake de Vlas
Henry de Vries
Natalie Dean
Kari Debbink
Mustapha Debboun
Amanda Debes
Anjan Debnath
Stijn Deborggraeve
Alain Debrabant
Alexander Debrah
Herbert De'Broski
Jacqueline Deen
Victor DeFilippis
Rosa Maria del Angel
Victor Del Rio Vilas
Eric Delaporte
Eric Delmelle
Benoit Dembele
Margriet den Boer
Matthew Denwood
Arminder Deol
Peter Deplazes
Kebede Deribe
Amare Deribew
David Deshazer
Alain Dessein
Gregor Devine
Angela Devine
Caroline Dewar
Ranadhir Dey
Javier Di Noia
Diawo Diallo
Michael Diamond
Luciane Dias-Melicio
Aissatou Diawara
Antonino Dicaro
Sean Diehl
George Dimopoulos
Diwakar Dinesh
Navid Dinparast Djadid
Ermias Diro
Erica Diruggiero
Colette Dissous
Sabine Dittrich
Maria Diuk-Wasser
Hazel Dockrell
Johannes Doehl
Norman Doggett
Esteban Domingo
Paola Dominici
Tiago Donatelli Serafim
Ke Dong
Marisa Donnelly

Jeffrey Donowitz
Amin Doosti Irani
Pierre Dorny
Adriana dos Santos
Reena Doshi
Annette Dougall
Richard Douglass
Kimberly Dowd
Philip Downs
Stephen Doyle
John Drake
Christopher Drakeley
Mike Drebot
Michael Drebot
Robert Dreibelbis
Paul Dresch
Mark Drew
Jan Felix Drexler
Anou Dreyfus
Anuradha Dube
Vikash Dubey
Audrey Dubot-Pérès
Breck Duerkop
Peter Dukes
Eric Dumonteil
Susanna Dunachie
Mike Dunbar
Jake Dunning
Veasna Duong
Salome Dürr
Georg Duscher
Jonathan Dushoff
Shanta Dutta
Gregory Ebel
Greg Ebert
Hideki Ebihara
Juan Echevarria
Shigetoshi Eda
Michael Eddleston
Robert Edelman
Anne Eder
Dileepa Ediriweera
W. John Edmunds
Thomas Edwards
Andrea Egizi
Lloyd Einsiedel
Alvaro Eiras
Obinna Ekwunife
Carole Eldin
Elizabeth Elhassan
Abeer El-Henawy
Celina Elissondo
Ivo Elliott
Alison Elliott
James Ellison
Nelly El-Sakkary
Najib El-Sayed
Paul Emerson
Samantha Emery
Nancy Endersby
Timothy Endy
David Engman
Christian Engwerda
Keeseon Eom
Astrid Erber
Marina Eremeeva
Koray Ergunay
Bobbie Erickson
Annette Ernhart
Kacey Ernst
Hildegund Ertl
Eugene Erulu
Teresa Escalante
Kevin Escandón-Vargas
Luis Escobar
Evan Eskew
Guido Espana
Marcos Espinal
Danuza Esquenazi
Elizabet Estallo
Michelle Evans
Bart Everts
Sara Eyangoh
Massimo Fabiani
Luca Facchinelli
Helen Faddy

Ahmed Fahal
Anna-Bella Failloux
Joseph Fair
Keke Fairfax
Franco Falcone
Padraic Fallon
Hui Wen Fan
Chia Fan
Li-Qun Fang
Marcelo Fantappie
Ary Faraji
Nicolas Fasel
Majid Fasihi Harandi
Rachel Fearns
Nicholas Feasey
David Fegan
Heinz Feldmann
Leora Feldstein
Philip Felgner
Carl Feng
Michael Ferguson
Ildefonso Fernández-Salas
Ana Fernandez-Sesma
Andrêa Jacqueline Ferreira
Elizabeth Ferreira
Henrique Ferreira
Claudia Ferreira
Tiago Ferreira
Ludmila Ferreira
María Ferrer
Eric Fevre
Matt Field
Joshua Fierer
Katherine Figarella
John Figarola
Claudio Figueira
Ulrike Fillinger
Paul Fine
Ashley Fink
Katja Fink
M. Firestone
Cadhla Firth
Roser Fisa
Philip Fischer
Marc Fischer
Rebecca Fischer
Peter Fischer
William Fischer
Christopher Fitzpatrick
Colin Fitzsimmons
Agnes Fleury
Ana Flisser
Adriana Flores
Monica Florin-Christensen
Robin Flynn
Dina Fonseca
Albin Fontaine
Luis Fonte
Anthony Fooks
Pierre Formenty
Naomi Forrester
Brett Forshey
Geraldine Foster
Florence Fouque
Kimberley Fox
Leanne Fox
Jay Fox
Betsy Foxman
Brian Foy
Deborah Fraga
Jose R. Franco
Jose Franco
Heitor Franco de Andrade
Carlos Franco-Paredes
Richard Franka
Richard Franka
David Freedman
Alexander Freiberg
Célio Freire-de-Lima
Moises Freitas
Michael French
Patrick French
Manuel Fresno
Conrad Freuling
Heather Friberg
Megan Fritz

Samuel Fuhrimann
Paula Fujiwara
Isaac Chun-Hai Fung
Luis Furuya-Kanamori
Sarah Gabriel
Albis-Francesco Gabrielli
Endalamaw Gadisa
Kenneth Gage
Montserrat Gállego
Manoj Gambhir
Dionicia Gamboa
Ekambaram Ganapathy
Sreenivas Gannavaram
Christian Ganoza
Rebecca Garabed
Teresa Garate
Melissa Garcia
Juan-Carlos Garcia R.
Manuel Garcia-Herranz
Jose Garcis-Bustos
Allison Gardner
Joshua Garn
Denise Garrett
Philippe Gasque
Katherine Gass
Jillian Gauld
Michael Gaunt
Bommakanti Gayathri
Soraya Gaze
Pedro Gazzinelli-Guimarães
Teshome Gebre
Stefan Geiger
Annemieke Geluk
Nicholas Generous
Giuilo Genovese
Fernando Genta
Christine Marie George
Antoine Gessain
Lorenzo Giacani
Malick Gibani
Wendy Gibson
Andrew Gibson
Lázaro Gil
Amy Gilbert
Tom Gilbert
Christina Gill
Lars Gille
John Gilleard
Robert Gilman
Geoffrey Gimonneau
Michael Ginger
Emilie Giraud
Patrick Giraudoux
Núria Gironès
Gregory Glass
Andrea Gloria-Soria
Eva Gluenz
Judith Glynn
Clement Gnanadurai
Cyrille Goarant
Geoffrey Gobert
Jacques Godfroid
Marco Goeijenbier
Arthur Goff
Tony Goldberg
Allison Golden
Pablo Goldschmidt
Delia Goletti
Delia Goletti
Kenneth Gollob
Bruno Gomes
Ciro Gomes
Maria Gomes-Solecki
Maria Adelaida Gomez
Ricardo Gomez
Beatriz Gómez
Maria Angeles Gomez Morales
Lígia Gonçalves
Celia Maria Gontijo
Jorge Gonzalez
Armando Gonzalez
Lilia Gonzalez-Ceron
Douglas Goodin
Alexander Gorbalenya
Catherine Gordon
Stephen Gordon

Ulla Gordon
Marga Goris
David Gorla
Maureen Gorman
Roly Gosling
Masamichi Goto
Hiro Goto
Nicole Gottdenker
Vanesa Gottifredi
Rene Gottschalk
Clément Goubert
Benjamin Gourbal
Sebastien Gourbiere
Anouk Gouvras
Giriyanna Gowda
Rodolphe Gozlan
Dennis Grab
Delia Grace
Luigi Gradoni
Loren Gragert
Jay Graham
Matthew Graham
Brian Graham
John Graham-Brown
John Grainger
Marina Gramiccia
Rawlin Grant
Warwick Grant
Nicholas Grassly
Berta Grau
Stephen Graves
Patricia Graves
Pascal Grebaut
Patrick Green
Christopher Gregory
Richard Grencis
Frederic Grenouillet
Helena Greter
Christoph Grevelding
Paul Griffin
Michael Griffiths
Elena Grigorenko
Mario Grijalva
Maria Eugenia Grillet
Jack Grimes
Alex Grinberg
Suzanne Groah
Gregory Gromowski
Martin Groschup
Jacques Grosset
Elysse Grossi-Soyster
Nathan Grubaugh
Christoph Grunau
Sarah Anne Guagliardo
Alessandra Guarneri
Jonathan Gubbay
Jean-Francois Guegan
Marcela Guerendiain
Pablo Guerenstein
Patrick Guerin
Richard Guerrant
Felipe Guhl
Luiz Guimarães
Ikram Guizani
Sharmini Gunawardena
Emily Gurley
Kyle Gustafson
José Gutiérrez
Julie Gutman
Pradip Gyawali
Theresa Gyorkos
Miklós Gyuranecz
Karen Haag
Jurgen Haanstra
Abdulrazaq Habib
Esmael Habtamu
Nabil Haddad
Andrew Haddow
Emily Hagan
Asrat Hailu
Homa Hajjaran
Julia Halder
Andrew Hall
Roy Hall
Katherine Halliday
Sonja Hall-Mendelin

Scott Halstead
Shinjiro Hamano
Gabriel Hamer
Sarah Hamer
Nicholas Hamm
Camila Hamond
Katie Hampson
Stefania Hanau
Patrick Hanington
Kathryn Hanley
Immo Hansen
Yuka Hara
Omar Harb
John Hargrove
Jonathan Harle
Gundel Harms
Billy Harnett
Julie Harris
Nicola Harris
Angela Harris
Jason Harris
Amy Hartman
Daiane Hartwig
Hideo Hasegawa
Masahiro Hashizume
Epco Hasker
Latiffah Hassan
Marisa Hast
Michelle Hawkins
Bill Hawley
Roderick Hay
Mary Hayden
Kenneth Hayes
Biao He
James Heffelfinger
Eva Heinz
Mark Heise
Jane Heller
Sarah Hendrickx
Debroski Herbert
Alexander Herbig
Cecile Hermann
Victor Herrera
Barbara Herwaldt
Jorg Heukelbach
Volker Heussler
Roger Hewson
Robert Heyderman
Leonard Heyerdahl
Matthew Higgins
Elizabeth Higgs
Eberhard Hildt
B. Joseph Hinnebusch
Jay Hinton
Kenji Hirayama
Alec Hirsch
Natasha Hochberg
Mary Hodges
Wayne Hodgson
Stephen Hoffman
Andreas Hofmann
Michael Hofreiter
Cornelis Hokke
Michael Holbrook
Lindy Holden-Dye
T Deirdre Hollingsworth
Edward Holmes
Sung-Jong Hong
Sung-Tae Hong
Nildimar Honorio
Edward Hook
Douglas Hooper
Jay Hooper
Pamela Hooper
D. Craig Hooper
Adrian Hopkins
John Horton
Daniel Horton
Duane Hospenthal
Wei-Cheng Hsu
Rongliang Hu
Victor Hu
Wenbiao Hu
Jian Huang
Yan-Jang Huang
Yu-Tung Huang

Xiaowen Huang
Marc Hubner
Marc Hübner
Hartwig Huemer
Jasmin Hufschmid
Grant Hughes
Leon Hugo
Noah Hull
John Humphrey
Tricia Humphreys
Debbie Humphries
Judith Humphries
Elizabeth Hunsperger
Peter Hunt
James Hurley
Stephen Hyslop
Ibtihal Mohamed Aly Ibrahim Abdel Rahman
John Ice
Alexander Idnurm
Valerio Iebba
Eliane Ignotti
Tetsuro Ikegami
Jung Ilhyo
Sanja Ilic
Ximena Illarramendi
Justin Im
Natsuko Imai
Jean-Luc Imler
Rubina Imtiaz
Timothy Inglis
Michael Irvine
Lourdes Isaac
N. A. Ismail
Victor Issa
Tadashi Itagaki
Masako Iwanaga
Anne Jaaskelainen
Alan Jackson
Yves Jackson
Shevin Jacob
Michael Jacobs
Robert Jacobs
Marc Jacobsen
Steven Jacobson
James Janetka
Olamide Jarrett
Edward Jarroll
Amelia Jazwa
Isabelle Jeanne
Selma Jeronimo
Aaron Jex
Tie-Wu Jia
Rays Jiang
Yongjun Jiao
Mark Jit
Asgeir Johannessen
Denny John
Shylo Johnson
Christian Johnson
Paul Johnson
Simon Johnston
Emmitt Jolly
Kathryn Jones
Malcolm Jones
Claire Jones
Douglas Jones
Kelsey Jones
Heesoo Joo
Heather Jordan
Marco José
Serene Joseph
Vinod Joshi
Peter Jourdan
Steven Juliano
Thomas Junghanss
Amy Junnila
Judith Justice
Malika Kachani
Tamilarasu Kadhiravan
Rebekah Kading
Siripen Kalayanarooj
Basile Kamgang
Joseph Kamgno
Seokyoung Kang
Seokyoung Kang

Vinicius Kannen
Chen-Yu Kao
Ray Kaplan
Evdokia Karagouni
Axel Karger
Samuel Kariuki
Stephan Karl
Erik Karlsson
Nadira Karunaweera
Shinji Kasai
Martin Kasny
Moses Katabarwa
Anne Katahoire
Vishwa Katoch
Alan Katz
Leah Katzelnick
David Kavili
Paul Kaye
Karen Keddy
Lindsay Keegan
Jeremy Keenan
Rebekah Keesler
Christian Keller
Paul Kelly
Alan Kemp
Volkhard Kempf
Jonathan Kennedy
Joanie Kenney
Peter Kern
Malgorzata Kesik-Brodacka
Jay Keystone
Amir Khan
Mohammad Khan
Siraj Khan
Farhana Khanam
Rajiv Khandekar
Rudo Kieft
Mariana Kikuti
Dong-Hyun Kim
Sunjung Kim
Christopher King
Uriel Kitron
Ute Klarmann-Schulz
Thomas Klei
Michael Klemba
John Klena
William Klimstra
Dan Kline
Jonas Klingström
Amy Klion
Johannes Klompen
Stephen Klotz
Susanne Kluh
Jenny Knapp
Barbara Knust
Gary Kobinger
Dhanpat Kochar
Anson Koehler
Julia Koehler
Cristian Koepfli
Alain Kohl
Bart Kooi
Kimberly Koporc
Pasi Korhonen
Poonum Korpe
Titia Kortbeek
Jeroen Kortekaas
Sudeep Kothari
Linda Kothera
Benjamin Koudou
Artemis Koukounari
Thomas Kozel
Uriel Koziol
Lukasz Kozubowski
Moritz Kraemer
Alexander Kraemer
Lutz Krause
Peter Krause
Nancy Krebs
Alison Krentel
Marco Krieger
Natraj Krishnan
Suma Krishnasastry
Mojca Kristan
Igor Krizaj
Axel Kroeger

Alejandro Krolewiecki
P. Kropf
Jürgen Krücken
Andreas Krüger
Ralf Krumkamp
Hilde Kruse
Adam Kucharski
Annette Kuesel
Senanayake Kularatne
Takashi Kumagai
Ambuj Kumar
Sanjai Kumar
Dibyendu Kumar
Devender Kumar
Roland Kupka
Tim Kurtti
Ivan Kuzmin
Natalia Kuzmina
Andreas Kuznik
Jin-Woo Kwon
Marcelo Labruna
Juan Laclette
Shannon LaDeau
Monique Lafon
Eric Lafontaine
Ramanuj Lahiri
Chih-Cheng Lai
Pritesh Lalwani
Poppy Lamberton
Louis Lambrechts
Claudio Lanata
Fiona Lange
Felix Lankester
Alexander Lankowski
Dhafer Laouini
Pascal Lapierre
Vicente Larraga
Sébastien Larréché
Robert Lash
Edwin Lasonder
Colleen Lau
Eric Lau
Susana Laucella
Stephen Lauer
Miriam Laufer
Volker Lauschke
Andreas Laustsen
Glenn Laverack
Anne Lavergne
Catherine Lawrence
Helen Lazear
Claudio Lazzari
Thanh Hoa Le
Monique Léchenne
Luis Lecuona
Rogan Lee
Jay Lee
Mariska Leeflang
Rosemary Lees
Shelley Lees
Joceline Lega
Pedro Legua
Linda Lehman
Andrew Leidner
Gustavo Leite
Veerle Lejon
Elba Lemos
Florian Lempp
Yee-Sin Leo
Tomas Leon
Tomas Leon
Guillermo Leon
Elli Leontsini
Stije Leopold
Louis-Philippe Leroux
Andres Lescano
William Letson
Rob Leurs
Bruno Levecke
Michael Levin
Myron Levine
Rebecca Levine
Sheri Lewis
Joseph Lewnard
Sheng Li
Yuan Li

Jun Li
Ya-Pin Li
Qisheng Li
Jianyong Li
Daniel Libraty
Thomas Lietman
Adrian Lifschitz
Marshal Lightowlers
Walter Lilenbaum
Adam Lima
Aldo Lima
Direk Limmathurotsakul
Zhonghui Lin
Yi-Ling Lin
Jessica Lin
Xiaorong Lin
Margaret Lind
John Lindo
José Angelo Lindoso
Steven Lindsay
Jo Lines
W. Ian Lipkin
Qin Liu
Wei Liu
Kangkang Liu
Alejandro Llanos
Manuel Llinás
Anne Lockyer
Philipe Loiseau
P'ng Loke
Eric Lombardini
Fabrizio Lombardo
Bruno Lomonte
Berlin Londono
Kanya Long
Silvia Longhi
Rhea Longley
Damaris Lopera
Job Lopez
Jennifer Lord
Andrea Lorden
Marcelo Lorenzo
J. Lorenzo-Morales
Maria Lorono-Pino
Felix Lötsch
Leon P. Lounibos
Flávio Loures
Philip LoVerde
Nicola Low
Jenny Low
Rachel Lowe
Andre Loxton
Matthew Lozier
Yoel Lubell
Stephen Luby
Juan Ludert
Julius Lukes
Lucy Lum
Jan Lundstrom
Britta Lundström-Stadelmann
Richard Luque
Francisco Luquero
Mimi Lusli
Sara Lustigman
Gareth Lycett
Gareth Lycett
Penny Lynch
Stacey Lynch
Wenjun Ma
David Mabey
Paulo Roberto Machado
Fabiana Machado
Carlos Machain-Williams
Fernando Macian
Charles MacKenzie
Stephen Mackessy
Tim Mackey
Annette MacLeod
Colin Macleod
Ken Maeda
Margaret Mafe
Tereza Magalhaes
Isabelle Magalhaes
Sadiqa Mahmood
Carla Maia
Bradley Main

Eimorn Mairiang
Rick Maizels
Ceila Malaque
Ceila Malaque
Gathsaurie Malavige
Emilio Malchiodi
Rosa Maldonado
Mwele Malecela
Wanchai Maleewong
John Malone
Mark Manary
Christian Mandl
Reeta Mani
Brian Mann
Rebeca Manning
Carrie Manore
Pablo Manrique Saide
Pablo Manrique-Saide
Ben Mans
Dennis Mans
Sunny Mante
Georges Mantion
Dorit Maoz
Pablo Maravilla
Paula Marcet
Jonathan Marchant
Antonio Marcilla
Ivan Marcipar
Elaine Marcos
Lorenzo Mari
Almudena Mari Saez
Mara Mariconti
Simon Mariwah
Wanda Markotter
Michael Marks
Florian Marks
Alexandre Marques
Wilson Marques Jr.
Mark Marsh
Fabiola Martin
Estelle Martin
Javier Martin
Fred Martineau
Juan Martinez
Mônica Martins
Ademir Martins
Gerald Marx
Andrea Marzi
Mauro Marzochi
Pascal Mäser
Daniel Masiga
Dmitri Maslov
Carl Mason
Alessandro Massolo
Pietro Mastroeni
Laura Mate
Waldor Mathew
Anuja Mathew
Derrick Mathias
Greg Matlashewski
Richard Maude
Aaron Maule
Isabel Mauricio
Wendy Maury
Stephanie Mauti
Alfredo Mayor
Humphrey Mazigo
Katarzyna Mazur-Melewska
Pamela Mbabazi
Evaristus Mbanefo
Gerald Mboowa
Monica McArthur
Laura-Isobel McCall
John McCreadie
Marianea McDonald
Juan McEwen
Johnjoe McFadden
Emma McIntosh
Heather McKay
Rima McLeod
Chris McMahan
W. Robert McMaster
Alan McNally
Darlene McNaughton
Paul McVeigh
Takafira Mduluza

Guruprasad Medigeshi
Graham Medley
Terri Meinking
Leonardo Melchior
Breno Mello
Ana Melo
Tiago Mendes
Jairo Mendez-Rico
Claudio Meneses
Cristiane Menezes
Cristiane Menezes
Joris Menten
Andres Merits
François-Xavier Meslin
Louisa Messenger
Sharon Messenger
Lynne Messer
Stefan Metz
David Meya
Hermann Meyer
Kristin Michel
Paul Michels
Jo Middleton
Janet Midega
Victor Midlej
Nicholas Midzi
Liesbeth Mieras
Luis Mier-y-Teran
Andrei Mihalca
Sebastian Mikolajczak
Anita Milicic
Gabriel Milinovich
W. Allen Miller
Louis Miller
Jonathan Miner
Corrado Minetti
Eric Mintz
Marcelo Mira
Mary Elizabeth Miranda
M. E. Miranda
Chad Mire
Paul Mireji
Andrew Mirelman
John Misasi
Richa Misra
Piers Mitchell
Edward Mitre
Yuji Miyamoto
Gerald Mkoji
Farrokh Modabber
Cassie Modahl
David Modrý
Israel Molina
Alvaro Molina-Cruz
Marlo Möller
David Molyneux
Federica Monaco
Dinesh Mondal
Luke Mondor
Karina Mondragon-Shem
Juthathip Mongkolsapaya
Carlos Montanari
Juan Antonio Montaño-Hirose
Wuelton Monteiro
Susan Montgomery
Rafael Montiel
Antonio Montresor
David Moo Llanes
Sean Moore
Susan Moore
Susan Moore
Serge Morand
Nicole Moreland
Silvia Moreno
Max Moreno Madriñán
Fernanda Morgado
Thomas Morgan
Tara Moriarty
Ignacio Moriyon
Ryan Mork
Liam Morrison
Amy Morrison
Thomas Morrison
Renato Mortara
Lydia Mosi
Sarah Moss

David Mosser
Licia Mota
Vladimir Motin
Jeremy Mottram
Gastón Adolfo Mougabure Cueto
Lawrence Moulton
Maria Paula Mourão
Luz Moyano
Caleb Mpyet
Ivo Mueller
Allan Muhwezi
Zindoga Mukandavire
Samson Mukaratirwa
Grace Mulcahy
Thomas Müller
Pie Müller
Ingrid Müller
Beat Müllhaupt
Kristin Mullins
John Mumford
Hetron Munang'andu
Ulrike Munderloh
Cesar Munoz-Fontela
Michele Murdoch
Bernadette Murgue
Gerald L. Murray
Dj Murry
Silvane Murta
Baba Musa
Anwar Musah
Carlos Muskus
John-Paul Mutebi
Ngoy Mutombo
Ankur Mutreja
Ephantus Muturi
Adrian Muwonge
Elibariki Mwakapeje
Jonathan Mwangi
Peter Myler
Ben Naafs
Helena Nader
Susan Nadin-Davis
Jyothi Nagajyothi
Rana Nagarkatti
Yoshinori Nakazawa
Hira Nakhasi
Emmanuel Nakoune
Jarlath Nally
Vu Nam
Sorrel Namaste
Mark Nanyingi
Miriam Nanyunja
Kaabia Naoufel
Ashwath Narayana
Dean Nardelli
Osvaldo Nascimento
Bruno Nascimento
Stephen Nash
Theodore Nash
Jim Nataro
Indira Nath
Avindra Nath
Sheila Nathan
Sheila Nathan
Norman Nausch
Severine Navarro
Miguel Navarro
Julius Ndukum
Girish Neelakanta
Fábio Negrão
Francesco Negro
Henok Negussie
Daniel Neill
Aaron Neiman
L. H. Nel
Andrew Nelson
Eric Nelson
Malden Nesheim
Aaron Neumann
Christoph Neumann Haefelin
Andreas Neumayr
Ana Gisele Neves-Ferreira
Irene Newton
Paul Newton
Johan Neyts
Nguyet Nguyen

Augusto Nhabomba
Peter Nicholls
Mark Nichter
André Nicola
Matthias Niedrig
Kirsten Nielsen
William Nierman
Nathan Nieto
Natalie Nieuwenhuizen
Masaaki Niino
Birgit Nikolay
Birgit Nikolay
Roshan Niloofa
Alasdair Nisbet
Sergio Nishioka
Andreas Nitsche
Zablon Njiru
Doris Njomo
Joo Hwan No
Aline Nobre
Takeshi Noda
Maurício Nogueira
Matthew Nolan
Gregory Noland
Navideh Noori
Douglas Norris
Steven Norris
Rosario Notaro
Norbert Nowotny
Belkisyole Noya
Elaine Nsoesie
João Nunes
Caris Nunes
Andrew Nuss
Thomas Nutman
Luke Nyakarahuka
Ruth Nyangacha
Gonzalo Obal
Clara Ocampo
Leon Ochiai
Josiah Ochieng
Eric Ochomo
Torsten Ochsenreiter
Peter Odermatt
Maurice Odiere
Nicholas Ogden
Stephanie Ogden
Myoung-Don Oh
Jun Ohashi
Kenji Ohnishi
Kamolnetr Okanurak
Joseph Okeibunor
Iruka Okeke
Chioma Okeoma
Collins Okoyo
Kendi Okuda
Oladimeji Oladepo
Gaetano Oliva
Clelia Oliva
Fabrício Marcus Oliveira
Ricardo Oliveira
Fabiano Oliveira
Piero Olliaro
Annette Olsen
Victoria Olson
Donald Olson
Michal Olszewski
Sandra O'Neill
Sheila Ons
Eng Eong Ooi
Kenneth Opara
Laor Orshan
Aaron Osgood-Zimmerman
Modupe Osinubi
Lyda Osorio
Domenico Otranto
Eric Ottesen
Jennifer Ottino
Marc Ouellette
Johnson Ouma
Paul Overgaauw
Katharine Owers
Arthur Owora
Yusuf Ozbel
Seray Özensoy Töz
Christopher Paddock

Wendy Page
Anne-Laure Page
Célia Pais
Claudia Paiva
Monica Pajuelo
Jennifer Palmer
Cody Palmer
Glen Palmer
Ramendra Pandey
Junxiong Pang
Igor Paploski
Peter Pappas
Nicholas Pardikes
Carlos Pardo
Luc Paris
Sangshin Park
Colin Parrish
Christopher Parry
Christopher M Parry
Marilyn Parsons
M. K. Parvez
Erica Pasini
Shital Patel
Bev Paterson
Beverly Paterson
Jean L. Patterson
Kimberly Paul
Richard Paul
Silke Paust
Patricia Pavlinac
Daniel Payne
Corey Peak
Richard Pearson
Mark Pearson
Samuel Peasah
André Pedrosa
Rosanna Peeling
Charlotte Peeters
Henry Peixoto
Jose Pelegrino
Herve Pelloux
Peter Pemberton-Ross
Xin Yu Peng
Johannes Penner
Pamela Pennington
Maria Grazia Pennisi
Claudio Pereira
Eugênia Pereira
Rushika Perera
Cecilia Perez Brandan
Alex Perkins
Guey Chuen Perng
Guey Chuen Perng
Donna Perry
Felipe Pessoa
Nathan Peters
Brian Peters
Jennifer Peterson
Townsend Peterson
Susan Petterson
Patrizio Pezzotti
Kenneth Pfarr
Matthew Phelps
Anitha Philbert
Anna Phillips
Derek Pickard
Harry Pickering
Brett Pickett
Raymond Pierce
Dennis Pierro
Gorben Pijlman
Allan Pillay
Nils Pilotte
Denise Pimenta
Jairo Pinheiro
Rosa Pinho
Clemencia Pinilla
Somchai Pinlaor
Amy Pinsent
C. Miguel Pinto
Vitor Pinto Junior
Sebastien Pion
Toni Piseddu
James Platts-Mills
Anita Plenge-Boenig
Gina Polo

Sittisede Polwiang
Christelle Pomares
Marco Pombi
Dinoop Poonambath
Stephen Popper
Sven Poppert
Travis Porco
Françoise Portaels
Marinete Povoa
William Powderly
Jeffrey Powell
Edoardo Pozio
Joaquín M. Prada
Vijay Prajapati
Clarissa Prazeres da Costa
Geoffrey Preidis
James Prendergast
Andrew Prendergast
Ric Price
Erin Price
Eric Prina
Gerardo Priotto
D. Pritchard
Anna Protasio
Rosana Puccia
Rosana Puccia
Juliet Pulliam
Narain Punjabi
Chaturong Putaporntip
Moneeb Qablan
Firdausi Qadri
Mengcen Qian
Cheng-Feng Qin
Juarez Antonio Quaresma
Rupert Quinnell
Juan Carlos Quintana-Castillo
Yvonne Qvarnstrom
Ana Rabello
Peter Rabinowitz
Abdul Rahman
Kazi Rahman
Antonio Raimundo Pinto de Almeida
Jahanavi Rajagopal
Ram Rajasekharan
Glenn Rall
Srinivasan Ramakrishnan
Sreejith Ramakrishnan
Srinivasan Ramakrishnan
Marcelo Ramalho-Ortigao
Kritika Ramani
Rajendranath Ramasawmy
Michael Ramharter
José Luis Ramirez
Juan David Ramírez
Juan Ramírez
Antônio Carlos Ramos
Alberto Ramos Jr.
Tommy Rampling
Michele Ramsay
Alice Ramyil
Nistara Randhawa
Hilary Ranson
Gordana Rasic
Kavi Ratanabanangkoon
Sumanta Ray
Simon Rayner
Julian Rayner
Jonathan Read
Paul Ready
Stanislas Rebaudet
Eduardo Rebollar-Tellez
Steven Reed
Erin Rees
Erin Rees
Rosa Reguera
Kathryn Reif
Lisa Reimer
Robert Reiner
William Reisen
William Reisen
Carolina Reisenman
Michael Reiskind
Paul Reiter
Ping Ren
Madhubala Rentala
Alfons Renz

Jose Requena
Ana Requena-Méndez
Rodrigo Resende
Antonio Ribeiro
Fatima Ribeiro-Dias
Flavia Ribeiro-Gomes
Frank Richards
Allen L. Richards
Jack Richards
Joshua Richardson
Jan Hendrik Richardus
Rebecca Rico-Hesse
Darren Riddy
Michael Riehle
Paula Rigato
Suman Rijal
Eleanor Riley
Gabriel Rinaldi
Björn Rissiek
Scott Ritchie
Koert Ritmeijer
Gilles Riveau
Jacob Riveron
Caitlin Rivers
Jo Robays
Chrissy Roberts
Sigrid Roberts
Lucy Robertson
Derrick Robinson
Mark Robinson
Arodys Robles
Manoel Otávio Rocha
Ilia Rochlin
Kat Rock
Barry Rockx
Isabel Roditi
Nilton Rodrigues
Rubens Rodriguez
Isabel Rodriguez-Barraquer
Peter Roeder
Matthew Rogers
Jason Rohr
David Roiz
Diana Rojas
Darin Rokyta
Kenneth Roland
David Rollinson
Katharina Röltgen
Danilo Roman-Campos
Patricia Romano
Gustavo Romero
Thomas Romig
Shannon Ronca
Lijun Rong
Pierre Roques
Bruce Rosa
Maria Goreti Rosa Freitas
Marcelo Rosandiski Lyra
Kerstin Rosenberger
Jason Rosenfeld
Martina Rothenbühler
Parimita Routray
Joel Rovnak
Chad Roy
Peter Roy
Dominik Rückerl
Claudia Rueckert
Pollie Rueda
Marie-Thérèse Ruf
Alessia Ruggieri
Estela Ruiz-Baca
José Antonio Ruiz-Postigo
Nick Ruktanonchai
Silvia Runge-Ranzinger
Colin Russell
Bruce Russell
Elmar Saathoff
Karla Saavedra-Rodriguez
Claude Sabeta
David Sack
David Sacks
Serge Alain Sadeuh-Mba
Fabian Saenz
David Safronetz
Bhaskar Saha
Sanjeev Sahni

Masayuki Saijo
Juan-Carlos Saiz
Naseem Salahuddin
Rehana Salam
Marcel Salathé
David Salazar-Valenzuela
Jorge Salinas
Henrik Salje
Jeanne Salje
Oscar Salomon
Kumar Sampath
D. Scott Samuels
John Samuelson
Abdallah Samy
Josemir Sander
Angelia Sanders
John Sanders
Rosemary Sang
Moussa Sanogo
Sonia Santibanez
Mauricio Santillana
Saul Santivanez
Marcos Santos
Renato Santos
Richard Sanya
Nancy Saravia
Kavitha Saravu
Bahador Sarkari
Abdur Sarker
Manoel Sarno
Euzenir Sarno
Elsa Sarti
Smitha Sasindran
Davide Sassera
Jetsumon Sattabongkot
Eleanor Saunders
Paul Saunderson
Stephen Savarino
Anthony Saviola
Lorenzo Savioli
C. E. Scantlebury
Vera Scarpassa
Samuel Scarpino
Samuel Scarpino
Fabian Schär
Birgit Schauer
Frieder Schaumburg
Robert Schaut
Sergio Schenkman
Philipp Scherer
John Schieffelin
Dieter Schifferli
Alejandro Schijman
Veronika Schmidt
Andre Schmidt
Veronika Schmidt
Thomas Schmidt
Achim Schnaufer
Matthias Schnell
Manuela Schnyder
Randal Schoepp
Steve Scholand
Tony Schountz
Albert Schriefer
Verena Schuenemann
Amy Schuh
Phillipp Schwabl
Tom Schwan
Eli Schwartz
Erich Schwarz
Jessica Schwind
David Scollard
Diana Scorpio
Pamela Scorza
Phillip Scott
Gavin Screaton
Nagila Secundino
Anthony Seddoh
Antonio Seguro
Jessica Seidman
Karin Seifert
Jorge Seixas
Stephen Seligman
Angamuthu Selvapandiyan
Paul Selzer
Roshanak Semnani

Debarka Sengupta
Mirgissa Serbessa
Denis Sereno
Esteban Serra
Solange Serrano
David Serre
Alexandre Servat
J. Seto
William Setzer
Anaiá Sevá
David Severson
Varadan Sevilimedu
Kairav J. Shah
Manjunath Shankar
Theresa Shapiro
Igor Sharakhov
Iraj Sharifi
Mansi Sharma
Yagya Sharma
Sanjib Sharma
Rahul Sharma
Tyler Sharp
L. S. Shashidhara
Alaullah Sheikh
Todd Shelper
Donald Shepard
William Shereni
Anita Shet
Pei-Yong Shi
Clive Shiff
Clive Shiff
Maria Aparecida Shikanai-Yasuda
Gabriel Shirima
Ziv Shkedy
Shmuel Shoham
Poojan Shrestha
Zhisheng Shuai
Keats Shwab
Beatriz Sierra
Ariel Silber
Erica Silberstein
Israel Silman
Adam Silumbwe
Ana Luiza Silva
Laurie Silva
Anjana Silva
José Franco Silveira
Judith Silverman
Shuzhen Sim
Rachel Simmonds
Cameron Simmons
Graham Simmons
Gustave Simo
Andrew Simpson
Calvin Sindato
Burton Singer
Rakesh Singh
Sarman Singh
Brajendra Singh
Ruchi Singh
Balbir Singh
Upinder Singh
Harinder Singh
Pushpendra Singh
Haruhiko Siomi
Andre Siqueira
Jair Siqueira-Neto
Amir Siraj
Amir Siraj
Padet Siriyasatien
Daouda Sissoko
Paiboon Sithithaworn
Patrick Skelly
Laura Skript
Jan Šlapeta
Dennis Slate
Barton Slatko
Sally Slavinski
Adrian Sleigh
Laurence Slutsker
David Smajs
Pieter Smit
Todd Smith
Rebecca Smith
Darci Smith
Peter Smith

David Smith
William Cairns Smith
Lucy Smith Paintain
Hermelijn Smits
Danielle Smyth
Georges Snounou
Neci Soares
Rodrigo Soares
Maria de Nazaré Soeiro
Mohd Sofian-Azirun
John Soghigian
Susanne Sokolow
Evgeni Sokurenko
Javier Solera
Anthony Solomon
Tom Solomon
Kateryna Soloviova
Daniel Sonenshine
Yajun Song
Lynn Soong
Ghislain Sopoh
Javier Sotillo
Rachida Soulaymani
Marc Souris
Giovane Sousa
Maria Sousa
Danielle Souza
Wayner Souza
Jayme Souza-Neto
Reinaldo Souza-Santos
John Spencer
Jessica Spengler
Natalie Spillman
Stanley Spinola
Christina Spiropoulou
Dave Spratt
Anon Srikiatkhachorn
Venkateswara Sripathi
Agnes Ssali
Colin Stack
Simona Stager
Hans-Christian Stahl
Lola Stamm
Duncan Steele
Anna-Sofie Steensgaard
Andrew Steer
Sasa Stefanic
Christina Steffen
Robert Steffen
Ivo Steinmetz
Olle Stendahl
John Stenos
Yvon Sterkers
Eleanore Sternberg
Jeremy Sternberg
Lori Stevens
Philip Stewart
Ymkje Stienstra
Benoit Stijlemans
Andrea Stiller
O. Stine
Timothy Stinear
Alexander Stockdale
Steven Stoddard
Marija Stojkovic
Wilma Stolk
Chris Stone
Russel Stothard
J. Russell Stothard
Daniel Streicker
Adrian Streit
Daniel Strickman
Andreas Stroehlein
Claudio Struchiner
Ashley Stycynski
Tianyun Su
José Antonio Suarez
Brian Suarez Mantilla
Mayte Suarez-Farinas
Jose Suaya
David Sullivan Jr.
Deborah Sumari
Irene Sumbele
Artur Summerfield
Jimin Sun
Wellington Sun

Piyanate Sunyakumthorn
Mehul Suthar
Tadaki Suzuki
Staffan Svard
Subramanian Swaminathan
Jonathan Swann
Earnest Tabah
John Tagg
Tahereh Taheri
Shannon Takala
Norihiro Takenouchi
Kawsar Talaat
Ernest Tambo
James Tamerius
Choo Hock Tan
Gladys Tan
Babasaheb Tandale
Mei San Tang
Sirikachorn Tangkawattana
Xiao-Yan Tao
Arnaud Tarantola
Rick Tarleton
Phillip Tarr
Rebecca Tarvin
Ann Tate
Daniel Tavares
Bineyam Taye
Jesse Taylor
Hugh Taylor
Louise Taylor
David Taylor-Robinson
Fabrizio Tediosi
Marcus Teixeira
Santuza Teixeira
Andréa Teixeira-Carvalho
Andrea Teixeira-Carvalho
Fasil Tekola-Ayele
Miriam Tendler
Jacob Tennessen
Olle Terenius
Luis Terrazas
Robert Terry
Kevin Tetteh
Janjira Thaipadungpanit
Saravanan Thangamani
Sally Theobald
Sophie Thevenon
Stephen Thomas
Rodolfo Thomé
Corinne Thompson
Sunnie Thompson
Panpim Thongsripong
S. Thumbi
Thorsten Thye
Huaiyu Tian
Michel Tibayrenc
Natalia Tiberti
Amanda Tiffany
Roger C. Tine
Yotsawan Tinikul
Daniel Tisch
Vasyl Tkach
Joao Toledo
Niraj Tolia
Abebayehu Tora
Noel Tordo
Paul Torgerson
Steve Torr
Alfredo Torres
José Tort
Pablo Tortosa
Jonathan Towner
Flavio Toxvaerd
Philip Toye
Yesim Tozan
Annelise Tran
Donato Traversa
Bruno Travi
Indi Trehan
Omar Triana-Chávez
Lucienne Tritten
Shaun Truelove
Richard Truman
Carine Truyens
Wen-Yang Tsai
Scheffer Tseng

Chien-Te Kent Tseng
Konstantin Tsetsarkin
Wuchun Tu
Apichai Tuanyok
James Tumwine
Joseph Turner
Hugo Turner
Paul Turner
Victoria Twort
Rebecca Tyson
Florencio Ubeira
Leonardo Uchiumi
Uade Ugbomoiko
Sebastian Ulbert
Buddy Ullman
Eduardo Undurraga
Joseph Urban
Julio Urbina
James Ussher
Nestor Uzcategui
Jonathan Vadnal
Patric Vaelli
Antti Vaheri
Hugo Valdivia
Vladimir Vale
Glyn Vale
Wim van Brakel
Govert van Dam
Andrew van den Hurk
Gert Van der Auwera
Wim van der Hoek
Tjip van der Werf
Angela van Diepen
Koenraad Van Doorslaer
Johan van Griensven
Henriette van Heerden
Lisette van Lieshout
Willem Van Panhuis
Nick Van Reet
Ronald Van Rij
Ronald Van Rij
Wesley Van Voorhis
Catiane Vander Kelen
Dana Vanlandingham
Kevin Vannella
Stefania Varani
Pedro Vasconcelos
José Ronnie Vasconcelos
Nikos Vasilakis
Jefferson Vaughan
Susana Vaz Nery
Gonzalo Vazquez-Prokopec
Sundeep Chaitanya Vedithi
Baruch Velan
Raman Velayudhan
Yael Velleman
Ramesh Venkatesh
Patricia Veras
Joey Verdi
Kristien Verdonck
Elisabeta Vergu
Sergio Verjovski-Almeida
Sten Vermund
Guilherme Verocai
Pauline Vetter
Agostinho Viana
Diego Viasus
Miguel Vicco
C. Rachel Vicente
Adriana Vicentini
Gema Vidal
Leda Vieira
Elvina Viennet
Marco Vigilato
Nicolas Vignier
Stalin Vilcarromero
Gustavo Vilchez
Sharon Yvette Angelina Villanueva
Luis Villar
Leopoldo Villegas
Marcos Villela
Marina Clare Vinaud
Joseph Vinetz
Marcus Virmond
Benjamin Jelle Visser
Simon Vitecek

Johnny Vlaminck
Chantal Vogels
Petr Volf
Veronika von Messling
Lorenz von Seidlein
John Vontas
Neil Vora
Adriaan Vos
Claire Waddington
David Wagner
Helene Wahlström
Diane Waku-Kouomou
Etienne Waleckx
Patricia Walker
Thomas Walker
Anthony Walker
Martin Walker
David Walker
Edward Walker
Ryan Wallace
Pegine Walrad
Judd Walson
Katharine Walter
Yvonne Walz
Li Wang
Zhuo Yu Wang
Lihua Wang
Lin-Fa Wang
Tianfang Wang
Seok Mui Wang
Yina Wang
Kinley Wangdi
Tri Wangrangsimakul
Samuel Wanji
Mark Wansbrough-Jones
Michael Ward
Michael Ward
Honorine Ward
Mary Warrell
Gideon Wasserberg
Marion Wassermann
Toshiki Watanabe
Conall Watson
Douglas Watts
Scott Weaver
Emily Webb
Friedemann Weber
Bonnie Webster
David Weetman
James Weger-Lucarelli
Peter Weina
Hana Weingartl
Philip Weinstein
Daniela Weiskopf
Louis Weiss
Christian Wejse
Melanie Wellington
Chad Wells
Oliverio Welsh
Bohai Wen
Tzai-Hung Wen
Guilherme Werneck
Guilherme Werneck
T. West
Sheila West
Sarah White
Julian White
Michael White
K. Andrew White
Laura White
A. White Jr.
Gudrun Wibbelt
Deepthi Wickremasinghe
Ryan Wiegand
W. Wiersinga
Paul Wigley
Lalith Wijedoru
Ananda Wijewickrama
Henry Wilde
Annelies Wilder Smith
Annelies Wilder-Smith
Patricia Wilkins
Annie Wilkinson
Diana Williams
Diana Williams
Holly Williams

Craig Williams
Hugh Willison
Bridget Wills
R. Wilson
William Wilson
Anthony Wilson
Peter Winch
Cheryl Winkler
Andrea Winkler
David Wohl
Timo Wolf
Adrian Wolstenholme
Kimberly Won
Kimberly Won
Charles Wondji
Patrick Woo
Se Joon Woo
James Wood
Caitlin Worrell
Jens Wrammert
Jans Wrammert
Joseph Wu
Zhongdao Wu
Hannah Wu
Xianfu Wu
Elsio Wunder Jr
Vanaporn Wuthiekanun
Tristram Wyatt
Susan Wyllie
Liyan Xi
Yanni Xiao
Cecilia Ximenez
Hong Xin
Xiaoning Xu
Hongwei Xu
Qin Xu
Chaoyang Xue
Sophie Yacoub
Zaida Yadon
Bereket Yakob
Kentaro Yamada
Hanano Yamada
Teresa Yamana
Thespina Yamanis
Yoshihisa Yamano
Gavin Yamey
Dan Yamin
Kun Yang
Chinglai Yang
Peizeng Yang
Zhicong Yang
Kun Yang
Richard Yapi
Emine Yaylali
Dorothy Yeboah-Manu
Tsin Yeo
Daniel Yeoh
Wenwu Yin
Samuel Yingst
In-Kyu Yoon
Nobuko Yoshida
Timothy Yoshino
Neil Young
Simon Young
Xue-Jie Yu
Farnaz Zahedifard
Mostafa Zamanian
Lorenzo Zammarchi
Paolo Zanotto
Herve Zeller
Bin Zhan
Liang Zhang
Xiang Zhang
Wenyi Zhang
Yaobi Zhang
Yong Zhang
Yanjin Zhang
Xingyu Zhang
Yae Zhao
Heping Zheng
Elyes Zhioua
Michael Zhiqiang Chen
Jianmin Zhong
Pingyu Zhou
Guan Zhu
Xiaofeng Zhu

Fabio Zicker
Emily Zielinski-Gutierrez
Eduard Zijlstra
Dan Zilberstein
Gert Zimmer
Sara Zimmer
Bianca Zingales
Jakob Zinsstag
Carina Zittra
Rachel Zufferey
Marcel Zwahlen
