# Supplementary material for: Correction: PLOS Neglected Tropical Diseases 2017 Reviewer and Editorial Board Thank You
Source: PLoS Negl Trop Dis. 2018 Apr 13;12(4):e0006424. doi: 10.1371/journal.pntd.0006424 (PMC5898705; doi:10.1371/journal.pntd.0006424)
Supplement: S1 Editor List — (PDF) [file pntd.0006424.s001.pdf]

PLOS Neglected Tropical Diseases would like to thank all those who served on the Editorial Board in 2017:

Alvaro Acosta-Serrano
Patricia V. Aguilar
Oladele B. Akogun
Serap Aksoy
Waleed Saleh Al-Salem
Benjamin Althouse
Andrew S. Azman
Robin L. Bailey
Stephen Baker
Anne-Laure Bañuls
Christopher M. Barker
Roberto Barrera
Alyssa E. Barry
María-Gloria Basáñez
Paul Andrew Bates
Daniel G. Bausch
David W. C. Beasley
Mark Quentin Benedict
Zvi Bentwich
Jeffrey Bethony
Alok Bhattacharya
Zulfiqar A. Bhutta
Philippe Billiald
Andrea Bingham
Brian Bird
David Blair
Marleen Boelaert
Mark Booth
Maria Elena Bottazzi
Klaus Brehm
Paul Brindley
Simon Brooker
Christine M. Budke
Barbara A. Burleigh
Carlos A. Buscaglia
Philippe Büscher
Alejandro Buschiazzo
Laurence U. Buxbaum
Adalgisa Caccone
Melissa J. Caimano
Guy Caljon
Cinzia Cantacessi
Michael Cappello
Hélène Carabin
Marilia Sá Carvalho
Edgar M. Carvalho
Nicholas R. Casewell
Jakob Zinsstag
Remi Charrel
Cheng-Chen Chen
Jean-Philippe Chippaux
Thomas S. Churcher
Archie Clements
Joachim Clos
Jenifer Coburn
Rita R. Colwell
Philip J. Cooper
Rodrigo Correa-Oliveira
Sergio Costa Oliveira
John Andrew Crump
John Pius Dalton
Stephen John Davies
Nicholas P. Day
Aravinda M. de Silva
Nilanthi de Silva
Janaka de Silva
Alain Debrabant
Hernando A. del Portillo
David Joseph Diemert
Rhoel Ramos Dinglasan
Mike J. Doenhoff
Eric Dumonteil
Walderez O. Dutra
Jan Dvorak
Daniel Eichinger
Uwem Friday Ekpo

Heidi G. Elmendorf
Christian Engwerda
Ananias A. Escalante
Marcelo U. Ferreira
Ana Flisser
Janet Foley
Matthew C. Freeman
Jennifer F. Friedman
Hans-Peter Fuehrer
Ricardo Toshio Fujiwara
Amadou Garba
Hector H. Garcia
Robin B. Gasser
Timothy G. Geary
Thomas Geisbert
Elodie Ghedin
Darren J. Gray
Robert M. Greenberg
Duane J. Gubler
Ricardo E. Gürtler
José María Gutiérrez
Maria G. Guzman
John Owusu Gyapong
Scott B. Halstead
David Harley
Eva Harris
Robert Harrison
Adrian B. Hehl
Kenji Hirayama
Achim Hoerauf
Olaf Horstick
Peter Hotez
Michael H. Hsieh
Jorge A. Huete-Pérez
Geoffrey K. Isbister
Akira Ito
Louise C. Ivers
Charles L. Jaffe
Anthony A. James
Armando Jardim
Aaron R. Jex
Michael A. Johansson
Malcolm Jones
Patricia Joyce
Narcis B. Kabatereine
Rebekah Crockett Kading
Shaden Kamhawi
Gagandeep Kang
Fatah Kashanchi
Matthew Kasper
Jennifer Keiser
Ben L. Kelly
Charles King
Kiyoshi Kita
Pattamaporn Kittayapong
Matty Knight
Stefanie Knopp
Albert Ko
Margaret Kosek
A. Desiree LaBeaud
David G. Lalloo
Patrick J. Lammie
Bruce Lee
Audrey Lenhart
Song Liang
Tao Lin
Alejandro Llanos-Cuentas
Diana N. J. Lockwood
Anuradha Lohia
James B. Lok
Benedito Antonio Lopes da Fonseca
Job E. Lopez
Hechmi Louzir
Sara Lustigman
Shan Lv
Kirsten E. Lyke
Andrew Scott MacDonald
Charles D. Mackenzie
Benjamin L. Makepeace
Ernesto T. A. Marques
Elizabeth Angelica Leme Martins
Daniel K. Masiga
Enock Matovu
Philip J. McCall
James S. McCarthy
Mary Ann McDowell
Anita K. McElroy
Rojelio Meija

Peter C. Melby
William B. Messer
Isaura Meza
Genevieve Milon
Makedonka Mitreva
Amy C. Morrison
Jorge Motta
Ana M. Moura-da-Silva
Dunstan Mukoko
Kosta Y. Mumcuoglu
Claudia Munoz-Zanzi
Grace Adira Murilla
Kristy O. Murray
Francisca Mutapi
Francis Mutuku
Pauline Mwinzi
Ana Nascimento
Joseph Mathu Ndung'u
Richard Ndyomugyenyi
Jeremiah M. Ngondi
Alessandra Nicoletti
Pedro L. Oliveira
Ken E. Olson
Scott L. O'Neill
Maria Victoria Periago
Christine A. Petersen
Richard Odame Phillips
Albert Picado
Mathieu Picardeau
Paulo Filemon Pimenta
Gerd Pluschke
Michael P. Pollastri
Ann M. Powers
Roger K. Prichard
Rachel L. Pullan
Sima Rafati
Didier Raoult
Jayne Raper
Jason L. Rasgon
Giovanna Raso
Sergio Recuenco
Richard Reithinger
Justin V. Remais
Todd B. Reynolds
Guilherme S. Ribeiro
José M. C. Ribeiro
Jessica N. Ricaldi
Rebecca Rico-Hesse
Ana Rodriguez
Alan L. Rothman
Charles E. Rupprecht
Edward T. Ryan
Reza Salavati
Rosemary C. Sang
Helton da Costa Santiago
Jose Ignacio Santos Preciado
Abhay R. Satoskar
Samuel V. Scarpino
Julius Schachter
Henk D. F. H. Schallig
Gabriele Schönian
W. Evan Secor
Abiola Senok
Edmund Y. W. Seto
Gary L. Simon
Steven M. Singer
Sunit Kumar Singh
Photini Sinnis
Pamela Small
Ricardo J. Soares Magalhaes
Philippe Solano
Sabine Specht
Banchob Sripa
Peter Steinmann
Francesca Tamarozzi
Herbert B. Tanowitz
Aysegul Taylan Ozkan
Louis-Albert Tchuem Tchuenté
Fasil Tekola Ayele
Robert B. Tesh
Yara M. Traub-Csekö
Christian Tschudi
Michael J. Turell
Jürg Utzinger
Jesus G. Valenzuela
Jan Van Den Abbeele
Joseph M. Vinetz
Judd L. Walson

Alon Warburg
Scott C. Weaver
Joanne P. Webster
Brian L. Weiss
Maya Williams
Elsio Wunder Jr.
Laith Yakob
Ruifu Yang
Guo-Jing Yang
Maria Yazdanbakhsh
Wenbao Zhang
Xiao-Nong Zhou
Jakob Zinsstag
